# Evaluation of Serum-Derived Bovine Immunoglobulin Protein Isolate in Subjects With Decompensated Cirrhosis With Ascites

**DOI:** 10.7759/cureus.15403

**Published:** 2021-06-02

**Authors:** Matthew J Stotts, Amanda Cheung, Muhammad B Hammami, David J Westrich, Eric Anderson, Lauren Counts, Alex S Befeler, Adrian M Di Bisceglie, Charlene Prather

**Affiliations:** 1 Gastroenterology and Hepatology, University of Virginia School of Medicine, Charlottesville, USA; 2 Division of Gastroenterology and Hepatology, Stanford University Medical Center, Stanford, USA; 3 Internal Medicine/Gastroenterology, University of California Riverside, Riverside, USA; 4 Department of Medicine, Saint Louis University School of Medicine, Saint Louis, USA; 5 Department of Internal Medicine, Washington University School of Medicine, Saint Louis, USA; 6 Department of Medicine, Saint Louis University, Saint Louis, USA; 7 Department of Gastroenterology and Hepatology, Saint Louis University School of Medicine, Saint Louis, USA

**Keywords:** bacterial translocation, chronic liver disease (cld), end-stage liver disease, bovine immunoglobulin, enteragam

## Abstract

Background

Bacterial translocation plays a pivotal role in the natural course of cirrhosis and its complications. Serum-derived bovine immunoglobulin (SBI) is an oral medical food that has been shown to both reduce inflammation in the intestines and neutralize bacteria. It represents a unique intervention that has not been studied in this population.

Methodology

We conducted a prospective open-label trial with an eight-week treatment phase of SBI. Individuals were assessed using lactulose breath testing, serum markers for enterocyte damage and bacterial translocation, and the Chronic Liver Disease Questionnaire (CLDQ) prior to and after completion of the treatment phase.

Results

We evaluated nine patients with a diagnosis of decompensated cirrhosis with ascites. Subjects had a mean Model for End-Stage Liver Disease (MELD) score of 11.6 ± 3.0 and were not taking lactulose or antibiotics. All subjects tolerated SBI well with no significant adverse events or changes to any of the six domains of the CLDQ. Laboratory tests including liver tests and MELD score remained stable over the course of treatment. There were no significant changes in the rates of small intestinal bacterial overgrowth (55.6% vs 55.6%, p = 1.00) or serum levels of lipopolysaccharide-binding protein, intestinal fatty acid-binding protein, or soluble CD14 (p-values 0.883, 0.765, and 0.748, respectively) when comparing values prior to and immediately after treatment.

Conclusions

No adverse events or significant changes to the quality of life were detected while on treatment. There were no statistically significant differences in our outcomes when comparing individuals before and after treatment in this small prospective proof-of-concept pilot study. Further prospective randomized studies could be beneficial.

## Introduction

The intestinal wall is a complex barrier that exists between humans and the environment. The commensal flora exposes the intestinal epithelium to nearly 100 trillion bacteria [1]. This epithelial layer provides a surface area of 400 square meters lined with tight junctions that prevent translocation and paracellular transport of luminal antigens including bacteria [2]. In addition to this mechanical barrier, the wall of the intestine is lined with mucosal immune defenses, notably gut-associated lymphoid tissue [1]. Under normal circumstances, this functional barrier effectively prevents the entry of bacteria from the outside world.

An increased rate of pathological bacterial translocation is associated with the development of decompensated cirrhosis and acute-on-chronic liver failure [3,4]. Contributing factors to bacterial translocation in cirrhotic patients include a higher prevalence of small intestinal bacterial overgrowth (SIBO) and impaired intestinal permeability as a result of widening of intercellular spaces, inflammation, and vascular congestion [3,5,6-8]. Failure of the intestinal barrier failure plays an important role in the natural course of cirrhosis, so much so that this has been referred to as hepatology’s “Achilles heel” [9]. In this setting, antibiotic prophylaxis has emerged as a widely accepted strategy [10]. The routine use of prophylactic antibiotics has led to the emergence of multi-resistant bacteria with increased mortality when these organisms become pathogenic [11]. Thus, alternative methods to prevent bacterial translocation in cirrhotic patients could be beneficial.

Immunoglobulins play a prominent role in health and development as demonstrated by the proven benefits of human breast milk and colostrum, a form of milk produced by mammals that contains significant amounts of immunoglobulins [12]. Recognition of the essential nature of these antibodies led to the development of commercial plasma-derived protein concentrates containing immunoglobulins, which have been used for decades in animal husbandry to promote growth and manage intestinal inflammation [13,14]. Similarly, studies in humans with various enteropathies have also shown beneficial effects on nutritional status and quality of life [15].

Serum-derived bovine immunoglobulin/protein isolate (SBI) is a novel medical food that is currently indicated for the clinical dietary management of several forms of enteropathy, including diarrhea-predominant irritable bowel syndrome and inflammatory bowel disease. While the term “enteropathy” refers to any pathology or disease of the intestines, known histological features can include blunting of intestinal villi, increased intraepithelial lymphocytes causing reduced absorptive capacity, and increased gut permeability [15]. In cases of enteropathy, a combination of factors including altered gut microbiota, increased intestinal inflammation, and worsening gut barrier dysfunction increases the risk of bacterial translocation [15]. Each of these factors is a potential target for SBI. In terms of altered gut microbiota, literature has demonstrated broad bacterial antigen neutralizing capacity of ingested immunoglobulins [16-18]. Likewise, many nonclinical studies have shown that SBI can reduce intestinal inflammation by decreasing mucosal cytokines and dampening immune activation [19,20]. Furthermore, the available data suggest that oral immunoglobulin therapy benefits tight-junction integrity in epithelial barriers, as evidenced through increased transepithelial electrical resistance and reduction in radiolabeled ^14^C-inulin permeability across the intestine [20].

To date, there is a large body of evidence showing that serum- or plasma-derived bovine immunoglobulin preparations can effectively manage the symptoms and harmful effects of enteropathy in both animals and humans. Animal studies in mice, rats, and pigs include data showing improved barrier function and nutrient absorption [15]. Studies performed in children show promising results in terms of weight gain and the underlying problem of malabsorption with relatively wide ranges in the duration studied (seven days to eight months) [21,22]. Among adults, preliminary studies show promising results of SBI in the management of HIV enteropathy in addition to diarrhea-predominant irritable bowel syndrome, with typical dosing of around 5 to 10 g daily and duration in the range of six to eight weeks [23,24]. Collectively, there is strong evidence to support the theory that supplementation with oral immunoglobulins such as SBI reduces the risk of bacterial translocation in patients with cirrhosis by neutralizing bacterial antigen in the intestine, reducing intestinal inflammation, and decreasing permeability of the gut barrier.

We hypothesize that individuals diagnosed with decompensated cirrhosis with ascites would have decreased rates of bacterial translocation when treated with SBI. The aim of our study was to assess the tolerability of SBI in individuals with cirrhosis, including the impact on their quality of life, as well as to quantify the impact of SBI on rates of SIBO and markers of bacterial translocation and gut barrier damage.

## Materials and methods

Study population

Adult male and nonpregnant females with a history of cirrhosis and ascites were considered eligible for this study. The diagnoses of cirrhosis and ascites were made by clinical and radiographical evidence. Exclusion criteria included recent bloodwork showing a Model for End-Stage Liver Disease (MELD) score of 17 or greater, history of transjugular intrahepatic portosystemic shunt placement, diagnosis of inflammatory bowel disease, pregnancy, hypersensitivity to bovine products, substance abuse, psychiatric disorders that would preclude the ability to complete the study, and use of lactulose or chronic antibiotics. The Institutional Review Board reviewed and approved the protocol (Clinical Trial Registry Number Identifier: NCT02608658). All subjects provided written informed consent. The American College of Gastroenterology funded this study.

Study design

This was a prospective, open-label, proof-of-concept, single-center pilot study performed in the outpatient setting. All participants in this study received 5 g of SBI (Enterahealth, Akeny, Iowa, USA) twice daily for eight weeks. Prior to the treatment phase, subjects were assessed in person. During this initial visit, blood samples were obtained, patients completed the Chronic Liver Disease Questionnaire (CLDQ), and a lactulose breath test (LBT) was performed. After four weeks, subjects were seen in person to assess compliance and record any adverse events. At the end of the study, subjects underwent repeat assessment identical to the initial visit. Results from the blood samples, CLDQ, and LBT were compared between the initial and last visit eight weeks later.

Study outcomes

The outcomes for this study included LBT for SIBO, markers of bacterial translocation and enterocyte damage including lipopolysaccharide-binding protein (LBP), soluble CD14 (sCD14), and intestinal fatty acid-binding protein (I-FABP), as well as changes in quality of life evaluated with the CDLQ.

LBT was performed using the standard protocol, including recommended fasting strategies and tobacco avoidance. Samples of end-expiratory air were collected every 20 minutes for 180 minutes after administration of 15 mL of lactulose diluted in water. Samples were analyzed using a Quintron Model SC gas chromatograph capable of measuring hydrogen, methane, and carbon dioxide. Results were determined as positive or negative based on the standard definitions of a positive breath test of either the presence of a “double peak” or a rise in breath hydrogen above 20 ppm within 90 minutes of lactulose ingestion.

The CLDQ was used to measure health-related quality of life, specifically focusing on the two weeks prior to the questionnaire administration. This instrument consists of 29 items compiled from six domains that include fatigue (five questions), abdominal symptoms (three questions), emotional function (eight questions), systemic symptoms (five questions), activity (three questions), and worry (five questions), and has been validated in several types of chronic liver diseases [25,26]. The responses for each question ranged from “all of the time” to “none of the time” and received a score ranging from 1 to 7, with higher scores indicative of better quality of life. Question responses were combined to be analyzed according to each domain.

LBP and sCD14 markers were used to evaluate for evidence of bacterial translocation, and I-FABP was measured to explore intestinal permeability. Plasma levels were measured using enzyme immunoassay for quantification according to the manufacturer’s recommendations (R&D Systems, Minneapolis, Minnesota, USA).

Safety assessment

Safety was assessed by conducting follow-up clinical visits with physical examinations and laboratory testing, including complete blood count, comprehensive metabolic panel, and prothrombin time at the initiation and completion of treatment. Adverse events were recorded for all subjects from the time of informed consent through the last dose of the investigational product.

Statistical methods

The following demographic and baseline characteristics were summarized descriptively using frequencies for categorical variables and means (with standard deviations [SDs]) for continuous variables: sex, race, age, and history of prior liver disease. Laboratory assessments including hematology, blood chemistry, and the aforementioned markers of bacterial translocation were summarized at baseline and at the end of the study. Laboratory values were summarized descriptively by mean, median, SD, and range. Paired t-tests were used to identify variables that were significantly different between pre- and post-intervention groups. Responses to the CLDQ questionnaire were reported as responses on a scale from 1 to 7 for each individual question and were summarized as mean and SD of all questions combined in each domain mentioned above. Paired t-tests were used to compare changes in the questionnaire responses from baseline and at the completion of the study. Comparisons between the pre- and post-intervention breath tests were described as positive or negative, with chi-square testing for significance. The statistical analysis was performed using STATA software (StataCorp, College Station, Texas, USA).

## Results

A total of 11 patients underwent formal enrollment. The median age was 59.9 (SD of 8.7). The two individuals who withdrew elected to stop the study shortly after enrollment and did not take any doses. Subjects were predominantly male (77.8%, n = 7) with causes of liver disease including nonalcoholic steatohepatitis (33.3%, n = 3), hepatitis C (22.2%, n = 2), alcohol (22.2%, n = 2), and hepatitis C/alcohol (22.2%, n = 2). Subjects had a mean MELD score of 11.6 (SD of 3.0, range 7 to 16). All individuals had a diagnosis of ascites and none were on antibiotics of any kind (including rifaximin or antibiotics for prophylactic reasons).

All individuals tolerated the SBI well with excellent compliance as determined at follow-up visits. No major or minor adverse events occurred during the study. There were no significant differences in individual labs, including serum sodium, creatinine, individual components of the hepatic function panel, platelets, or calculated MELD score before or after treatment (Table 1).

Serum-derived bovine immunoglobulin effect on the presence of bacterial overgrowth

Overall, 55.6% (5/9) of individuals tested positive for bacterial overgrowth on their initial LBT. After eight weeks of treatment with SBI, all individuals who tested positive on their initial assessment remained positive, and all of those who were negative on initial testing (44.4%, 4/9) remained negative at the end of treatment. In this setting, there was no statistical difference when comparing the pre-treatment and post-treatment groups (55.6% vs 55.6%, p-value = 1.0).

**Table 1:** Baseline and follow-up changes in individual labs and MELD score. All results are reported as mean (standard deviation). P-values were obtained by matched pair t-testing. MELD: Model for End-Stage Liver Disease

Laboratory variable	Pre-intervention [Mean (SD)]	Post-intervention [Mean (SD)]	P-value
Sodium (mEq/L)	137.1 (2.6)	137.2 (4.1)	0.95
Creatinine (mg/dL)	0.83 (0.2)	0.84 (0.2)	0.90
Total protein (g/dL)	7.84 (0.5)	7.60 (0.3)	0.24
Albumin (g/dL)	3.30 (0.3)	3.28 (0.4)	0.86
Bilirubin (mg/dL)	2.22 (1.08)	1.93 (0.9)	0.55
Alkaline phosphatase (IU/L)	118.8 (39.8)	106.2 (31.1)	0.47
Alanine aminotransferase (U/L)	46.1 (34.0)	32.9 (18.5)	0.32
Aspartate aminotransferase (U/L)	70.1 (62.7)	48.9 (20.2)	0.40
Platelets (per mcL)	95.6 (47.4)	85.8 (46.5)	0.67
MELD score	11.6 (3.0)	10.7 (2.6)	0.51

Serum-derived bovine immunoglobulin effect on markers of bacterial translocation and enterocyte damage

Circulating levels of LBP were similar at baseline compared to at the completion of the study (14.0 mg/mL, SD of 3.7 vs 13.7 mg/mL, SD of 0.8; p-value = 0.88). Similarly, no significant difference was seen in regards to I-FABP (3,529.3 pg/mL, SD of 1,244.8 vs 3,357.1 pg/mL, SD of 999.1; p-value = 0.76) and soluble CD14 (1,817.6, SD of 298.3 vs 1,869.0, SD of 1,590.6; p-value = 0.75) was appreciated.

Serum-derived bovine immunoglobulin effect on the quality of life

We assessed all patients for a baseline measure of quality of life using the CLDQ. We did not see any statistically significant differences in any of the six domains that were evaluated before and after treatment, including abdominal symptoms (5.74 vs 5.81; p = 0.87), fatigue (4.42 vs 4.29; p = 0.74), systemic symptoms (5.04 vs 5.00; p = 0.92), activity (5.44 vs 5.26; p = 0.74), emotional function (5.92 vs 5.42; p = 0.06), worry (5.16 vs 4.71; p = 0.24), or overall CLDQ score (5.31 vs 5.05; p = 0.12) (see Table 2).

**Table 2:** Baseline and follow-up changes in CLDQ scores. All results are reported as mean (standard deviation). P-values were obtained by matched pair t-testing. CLDQ: Chronic Liver Disease Questionnaire

CLDQ Domain	Baseline [Mean (SD)]	End of study [Mean (SD)]	P-value
Abdominal symptoms	5.74 (1.93)	5.81 (1.44)	0.87
Fatigue	4.42 (1.79)	4.29 (2.01)	0.74
Systemic symptoms	5.04 (2.24)	5.00 (1.78)	0.92
Activity	5.44 (2.24)	5.26 (1.81)	0.74
Emotional function	5.92 (1.47)	5.42 (1.68)	0.06
Worry	5.16 (1.71)	4.71 (1.82)	0.24
CLDQ overall score	5.31 (1.90)	5.05 (1.82)	0.12

## Discussion

This prospective open-label trial evaluated the use of SBI in individuals with decompensated cirrhosis with ascites. Subjects tolerated the therapy well, with all nine individuals completing the trial without significant adverse events or changes to any of the quality of life domains that we examined. Similarly, there were no concerns for progressive liver dysfunction in this group, as there were no significant changes in liver enzymes, bilirubin, albumin, sodium, and international normalized ratio when comparing individuals before and after treatment. There were no significant changes to the study outcomes of the presence of SIBO or markers of bacterial translocation.

Trials using SBI are currently underway for individuals with severe alcoholic hepatitis both in the United States and in India (NCT02265328, NCT01968382). However, there are no studies to date assessing the use of this medical food in individuals with advanced liver disease. Similar to decompensated cirrhosis of the liver, bacterial translocation is thought to play a major role in the disease course of alcoholic hepatitis and, as such, SBI represents a novel therapeutic approach that may prove effective. This is the first study evaluating the use of SBI in a cohort of subjects with advanced liver disease.

This study had several weaknesses that should be addressed. The duration of the study may have ultimately been too short to allow for physiologic changes. Given the nature of a pilot study, it was notably underpowered and part of this was due to difficulty in patient recruitment. The target population for this study was one that would be at high risk for bacterial translocation, particularly with portal hypertension as demonstrated by the presence of ascites. In clinical practice, many of these patients are on antibiotic therapy for SBP prophylaxis or they have concomitant hepatic encephalopathy that is treated with lactulose and/or rifaximin. However, the use of any of these medications including long-term antibiotic use were exclusion criteria because they would be confounding factors in the LBT results. Due to the lack of a control group, we could not exclude the possibility that the benefits of SBI were confounded by the natural progression of liver disease. Finally, while several potential surrogate markers for bacterial translocation have been proposed and studied for individuals with liver disease, there is currently no optimal biomarker for bacterial translocation that has been widely studied and validated in this patient population [27].

This is the first study evaluating the use of SBI in patients with decompensated cirrhosis or advanced liver disease. While there were no significant changes to markers of bacterial translocation or rates of SIBO, the use of SBI was tolerated well by the individuals in the study with no adverse events. Importantly, we also did not find any significant changes in quality of life over the course of eight weeks of treatment with this medical food, providing evidence that this product may be tolerated and safe for future prospective trials. Given the success of this medical food in other gastrointestinal disorders, including HIV enteropathy, inflammatory bowel disease, and irritable bowel syndrome, SBI remains an interesting intervention that could have a role in preventing bacterial translocation and its complications including spontaneous bacterial peritonitis, hepatic encephalopathy, hepatorenal syndrome, and infection in this population. Future prospective randomized studies are needed to prove benefit.

## Conclusions

Bacterial translocation across the intestinal epithelium is a known precipitant of clinical decompensation in individuals with advanced liver disease. In our small single-arm study, individuals with advanced liver disease tolerated SBI with no major changes to their quality of life or baseline tests of liver function. However, no major changes were seen in the rates of SIBO or markers of bacterial translocation. Further studies of this product in alcoholic hepatitis are underway, and additional prospective studies should be considered as SBI is a novel therapeutic intervention that may prove beneficial in this population.
